# Inactivation of *Acinetobacter baumannii* Biofilms on Polystyrene, Stainless Steel, and Urinary Catheters by Octenidine Dihydrochloride

**DOI:** 10.3389/fmicb.2016.00847

**Published:** 2016-06-09

**Authors:** Amoolya Narayanan, Meera S. Nair, Deepti P. Karumathil, Sangeetha A. Baskaran, Kumar Venkitanarayanan, Mary Anne Roshni Amalaradjou

**Affiliations:** ^1^Department of Psychological Sciences, University of Connecticut, StorrsCT, USA; ^2^Department of Animal Science, University of Connecticut, StorrsCT, USA; ^3^Department of Veterinary Public Health and Epidemiology, Veterinary College and Research InstituteThanjavur, India

**Keywords:** *Acinetobacter baumannii*, nosocomial infections, biofilm, octenidine dihydrochloride, stainless steel, urinary catheter

## Abstract

*Acinetobacter baumannii* is a major nosocomial pathogen causing human infections with significant mortality rates. In most cases, infections are acquired through exposure to *A. baumannii* biofilms that persist on contaminated hospital equipment and surfaces. Thus, it is imperative to develop effective measures for controlling *A. baumannii* biofilms in nosocomial settings. This study investigated the efficacy of octenidine dihydrochloride (OH), a new generation disinfectant for reducing *A. baumannii* biofilms on polystyrene, stainless steel and catheters. OH at 0.3% (5 mM), 0.6% (10 mM), and 0.9% (15 mM) was effective in significantly inactivating *A. baumannii* biofilms on all tested surfaces (*P* < 0.05). Furthermore, OH was equally effective in inactivating biofilms of multidrug resistant and drug susceptible *A. baumannii* isolates. In addition, confocal imaging revealed the predominance of dead cells in the OH-treated samples in comparison to the control. Further, scanning electron microscopy of biofilms formed on catheters revealed that OH treatment significantly reduced *A. baumannii* biofilm populations in corroboration with our antibiofilm assay. These data underscore the efficacy of OH in inactivating *A. baumannii* biofilms, thereby suggesting its potential use as a disinfectant or a catheter lock solution to control *A. baumannii* infections.

## Introduction

*Acinetobacter baumannii* is a Gram-negative opportunistic pathogen commonly associated with nosocomial infections (Peleg et al., 2008), including pneumonia, septicemia, wound infection, urinary tract infection, endocarditis, meningitis, blood stream infections, osteomyelitis, and corneal perforation (Bergogne-Bérézin and Towner, 1996; Ribera et al., 2003; Briggs et al., 2004; Dijkshoorn et al., 2007; Federico et al., 2011). It is ranked as one of the most common bacteria linked to infections associated with intensive care units (Giamarellou, 2010; Medell et al., 2013). *A. baumannii* has been identified as a “red alert” human pathogen, generating alarm among medical practitioners due to its extensive antibiotic resistance spectrum (Maragakis and Perl, 2008; Neonakis et al., 2011; Howard et al., 2012). The Infectious Diseases Society of America ranked *A. baumannii* as a top priority, antibiotic-resistant pathogens to target because of its rapid tendency to develop drug resistance, and the availability of a narrow choice of antibiotics to treat infections caused by the bacterium (Talbot et al., 2006).

Besides antibiotic resistance, a major concern with *A. baumannii* is its ability to form biofilms (Longo et al., 2014). Biofilms are surface-associated bacterial conglomerates that enable bacteria to survive and persist on abiotic surfaces for long periods of time while being resistant to commonly used antibiotics and disinfectants (Sambanthamoorthy et al., 2014). Several studies have demonstrated that biofilm formation permits the survival and persistence of *A. baumannii* on abiotic hospital surfaces resulting in tenacious and recurring nosocomial infections (Orsinger-Jacobsen et al., 2013; Longo et al., 2014; Sambanthamoorthy et al., 2014). Several antimicrobial candidates have been evaluated for their antibiofilm activity against *A. baumannii in vitro* and *in vivo*. These include antibiotics (Jamal et al., 2014; Wang et al., 2014), quorum sensing inhibitors, including lactonases and c-di-GMP inhibitors (Chow et al., 2014; Sambanthamoorthy et al., 2014), chimeric antimicrobial peptides (Gopal et al., 2014), antibiofilm peptides (Reffuveille et al., 2014), antimicrobial based wound gels (Gawande et al., 2014), disinfectants such as chlorhexidine, Lysol and bleach (Orsinger-Jacobsen et al., 2013) and other compounds like Virstatin, cerium, chitosan, and hamamelitannin (Cobrado et al., 2013; Chabane et al., 2014). Although these approaches have shown promise in the control of *Acinetobacter* biofilms, most have been investigated for application to prevent wound infections. However, it is well established that biofilm formation on abiotic surfaces such as medical devices plays a crucial role in the transmission of *A. baumannii* in nosocomial settings (Gaddy and Actis, 2009; McConnell et al., 2013). Therefore, besides controlling *Acinetobacter* associated pathologies, elimination of the pathogen from the hospital environment is equally critical to preventing *A. baumannii* infections in humans. Successful eradication of the pathogen from the nosocomial environment entitles the complete killing (inactivation) of the biofilm associated bacterial cells thereby rendering the biofilm dead and non-infectious.

Octenidine dihydrochloride (OH) is a positively charged bispyridinamine exhibiting antimicrobial activity against plaque-producing bacteria such as *Streptococcus mutans* and *S*. *sanguis* (Bailey et al., 1984). Recent studies have also demonstrated its antimicrobial efficacy against *Escherichia coli* O157:H7 (OH @ 0.15 and 0.25%), *Staphylococcus aureus*, Methicillin-resistant and Vancomycin-resistant *S. aureus* (MRSA, VRSA; OH @ 0.15, 0.3 and 0.6%), *Salmonella* Enteritidis (OH @ 0.15 and 0.25%), *Enterococcus faecalis* (OH @ 0.1%), *Candida albicans* (OH @ 0.1%) *Listeria monocytogenes* (OH @ 0.15 and 0.25%) and *Pseudomonas aeruginosa* (OH @ 0.1%; Zumbotel et al., 2009; Tirali et al., 2010; Baskaran et al., 2012; Amalaradjou and Venkitanarayanan, 2014; Junka et al., 2014; Makkar et al., 2015). OH was found to possess high tissue tolerability, and hence has been applied topically to prevent open wound infections (Hubner et al., 2010; Vanscheidt et al., 2012). Selcuk et al. (2012) demonstrated the antimicrobial efficacy of OH against *A. baumannii* in a mouse burn model, where they observed that wound treatment with octenisept (Schülke & Mayr GmBH, Norderstedt, Germany; active ingredient – 0.1% OH) significantly reduced *A. baumannii* populations in the eschar tissue compared to commonly used wound treatments such as silver sulfadiazine and mupirocin. Besides its antimicrobial effect, toxicity studies in a variety of species indicated that OH is not absorbed through the mucous membrane and gastrointestinal tract, with no reported carcinogenicity, genotoxicity or mutagenicity (Hirsch et al., 2009). However, no studies have investigated the antibiofilm effect of OH on *A. baumannii*, especially on surfaces commonly encountered in hospital settings. Therefore this study determined the efficacy of OH (0.3, 0.6, and 0.9%) to inactivate *A. baumannii* biofilms on polystyrene, stainless steel and urinary catheters.

## Materials and Methods

### Culture Preparation

All bacteriological media were purchased from Difco (Becton Dickinson, Sparks, MD, USA). The antibiofilm effect of OH was investigated using two *A. baumannii* isolates, namely *A. baumannii* ATCC 17978 (drug susceptible brain isolate, American Type Culture Collection, Manassas, VA, USA; Adams et al., 2009) and *A. baumannii* 190451 (multidrug resistant respiratory tract isolate, International health management associates, Schaumburg, IL, USA). The antibiotic resistance profile of *A. baumannii* 190451 was amikacin (MIC 64 μg/mL), amoxicillin (MIC 32 μg/mL), cefepime (MIC 32 μg/mL), ceftazidime (MIC 32 μg/mL), ceftriaxone (MIC 64 μg/mL), imipenem (MIC 4–32 μg/mL), levofloxacin (MIC 8 μg/mL), meropenem (MIC 16 μg/mL), minocycline (MIC 1–16 μg/mL), and piperacillin (MIC 128 μg/mL; Karumathil et al., 2014). Stock cultures were stored at -80°C in tryptic soy broth (TSB) with 50% glycerol. Prior to each experiment, each isolate of *A. baumannii* was grown individually on MDR *Acinetobacter* agar (Hardy Diagnostics, Santa Maria, CA, USA), and an individual colony from this medium was sub cultured at least three times in TSB for 24 h at 37°C with shaking (200 rpm). After the subcultures, bacterial cells were harvested from an overnight culture by centrifugation at 3,600 × *g* for 30 min at 4°C. The cells were washed twice in sterile phosphate buffered saline (PBS; 1x PBS pH 7.2 consisting of 137 mM NaCl, 2.7 mM KCl, 10 mM Na_2_HPO_4_, and 2 mM KH_2_PO_4_), and the bacterial cell pellet was finally resuspended in PBS, which was used as the inoculum. The bacterial population in the inoculum was confirmed by broth dilution and surface plating on tryptic soy agar (TSA) plates and incubation at 37°C for 24 h (Karumathil et al., 2014).

### Octenidine Dihydrochloride

Octenidine dihydrochloride (>99%) was obtained from Dishman USA, Middlesex, NJ, USA.

### Inactivation of *A. baumannii* Biofilms on Polystyrene by OH

The antibiofilm effect of OH was determined by microtiter plate assay (Amalaradjou et al., 2010). Briefly, *A. baumannii* cultures were separately grown overnight in TSB at 37°C. Following incubation, the cultures were sedimented by centrifugation (3,600 × *g* for 15 min), washed twice with PBS and resuspended in 10 mL of TSB. Two hundred microliters of the washed culture was used as the inoculum (∼6.0 log CFU). Sterile 96-well polystyrene tissue culture plates (Costar, Corning Incorporated, Corning, NY, USA) were inoculated with 200 μL of each bacterial cell suspension (∼6.0 log CFU) and incubated at 37°C for 24 h without agitation for biofilm production. Following biofilm formation, the effect of OH was tested at 0 (negative control), 0.3% (5 mM), 0.6% (10 mM), and 0.9% (15 mM) with an exposure time of 0, 1, 5, and 10 min. Since ethanol is a common disinfectant used in hospital settings and was used as the solvent for OH, all experiments also included samples that were treated with ethanol as a solvent control. After exposure to OH for the specified time, wells were washed three times with 200 μL of sterile PBS, and the adherent biofilm was scraped and plated directly or after serial dilution in PBS on TSA plates. The plates were incubated at 37°C for 24 h before enumerating biofilm-associated bacterial population. Duplicate wells were included for each treatment, and the assay was repeated three times.

### Biofilm Assay on Stainless Steel Matrix

Stainless steel (type 304 with a 4b finish) was used for making coupons (diameter: 1 cm). Stainless steel coupons were washed and cleaned prior to use, as described by Amalaradjou et al. (2009). *A. baumannii* cells were grown in TSB and diluted 1:40, as described before (Amalaradjou et al., 2009). Two hundred microliters of the inoculum were then dispensed onto the stainless steel coupons submerged in a 24-well plate (Becton Dickson Labware, Franklin Lakes, NJ, USA). Biofilm was formed at 37°C as before, and treated with 70% ethanol (solvent control), 0 (negative control), 0.3% (5 mM), 0.6% (10 mM), and 0.9% (15 mM) OH for 0, 1, 5, or 10 min. Following this, biofilm-associated *A. baumannii* cells were dispersed and enumerated (Ayebah et al., 2005). Duplicate coupons were included for each treatment, and the experiment was replicated three times.

### Biofilm Assay on Urinary Catheters

The efficacy of OH as a catheter lock solution ingredient in inactivating *A. baumannii* biofilms on catheters was determined according to a previously described protocol (Amalaradjou et al., 2010). A 12 F Foley urinary tract catheter (At Home Medical) was cut into 3-cm pieces. Each catheter piece was sealed at one end, filled with 1 mL of bacterial culture (∼6.0 log CFU) and sealed at the other end. The catheter pieces were then incubated at 37°C for 5 days to facilitate biofilm formation on the catheter lumen surface. After 5 days, each catheter piece was washed with sterile saline to remove unattached cells, sealed at one end, filled with 1 mL of sterile normal saline (negative control) or saline containing 70% ethanol (solvent control), 0.3% (5 mM), 0.6% (10 mM), and 0.9% (15 mM)OH, sealed at the other end and incubated at 37°C. The biofilm-associated bacterial population was determined following OH exposure (0, 15, 30, and 60 min) by enumerating bacteria after dislodging the biofilm from the catheter surface. This was achieved by vortexing the catheter pieces in separate tubes containing 10 mL of PBS for 1 min, followed by sonication at 40 KHz for 5 min in a bath sonicator (Branson, North Olmstead, OH, USA). After sonication, viable bacterial counts in PBS from each tube were enumerated after serial dilution (1:10 in PBS) and plating on duplicate TSA plates. Two catheter pieces were included for each treatment, and the experiment was repeated three times. In all the experiments, the antibiofilm effect of OH was also evaluated in the presence of serum albumin to simulate the presence of organic contaminants on hospital contact surfaces (Edmiston et al., 2006).

### Confocal Microscopy

*In situ* confocal laser scanning microscopy was done to visualize the efficacy of OH in inactivating *A. baumannii* biofilms (Amalaradjou and Venkitanarayanan, 2014). For microscopic assessment, *A. baumannii* biofilms were grown on Lab-Tech four-chamber borosilicate glass coverslip (Lab-tek, Nalge Nunc International, Rochester, NY, USA) at 37°C in TSB for 24 h. The microscopy was performed according to the method reported by Amalaradjou and Venkitanarayanan (2014). Briefly, preformed biofilms were treated with 0.6% (10 mM) OH for 60 min and the live and dead cells were imaged after staining with 2.5 μM SYTO (Molecular probes, OR) and 5 μM propidium iodide (PI, Molecular probes, OR). Biofilms not treated with OH were imaged to view the normal architecture of *A. baumannii* biofilm. Samples were examined under a Leica true confocal scanner SP2 microscope using the water immersion lens. A krypton-argon mixed gas laser with PMT2 filter served as the excitation source.

### Scanning Electron Microscopy (SEM)

*Acinetobacter baumannii* ATCC 17978 biofilm formation and inactivation by OH on stainless steel coupons and urinary catheters was examined by SEM (Djeribi et al., 2012). *A. baumannii* biofilms were formed on stainless steel coupons and catheter pieces as described previously. Following biofilm formation, the inoculated catheter pieces and coupons were washed with sterile PBS to dislodge the loosely attached and unattached bacterial cells. Subsequently, they were treated with either 0.6% (10 mM) OH or sterile PBS for 15 min at 37°C. Samples were then fixed in glutaraldehyde-paraformaldehyde-cacodylate buffer (pH 7) at 4°C for 90 min. Following fixation, catheters were washed with 0.1 M Na cacodylate buffer (pH 7) and post-fixed in 1% osmium tetroxide at 4°C overnight. The catheters were then rinsed twice for 15 min in distilled water, then dehydrated in serial concentrations of ethanol (30, 50, 70, 95, 100, and 100% ETOH, 15 min each), and critical point dried (931GL, Tousimis). The dried catheter samples were then mounted on SEM stub using silver paint and sputter coated with gold/palladium (E5100, Polaron) and examined using a scanning electron microscope (FEI Nova Nano SEM 450).

### Statistical Analysis

Duplicate samples were used for each treatment, and each experiment was replicated three times. For each treatment and control, data from independent replicate trials were pooled and analyzed using the proc GLM sub-routine of the statistical analysis software. The model included the treatment concentrations and time as the major effects. A least significant difference test was used to determine significant differences (*p* < 0.05) due to treatment concentrations and time on bacterial counts.

## Results and Discussion

The high nosocomial incidence of *A. baumannii* in endemic and epidemic situations can be attributed to their resistance to antibiotics and ability to form biofilms on hospital surfaces and medical devices (de Breij et al., 2010). Biofilm formation enhances *A. baumannii* colonization and persistence in the hospital environment, which in turn increases the likelihood of nosocomial infections (Espinal et al., 2012). Additionally, it has been demonstrated that biofilm formation on abiotic surfaces is a characteristic feature of clinical *A. baumannii* strains isolated from bloodstream infections and catheter-associated urinary tract infections (CAUTI, Feng et al., 2013). Therefore eradication of environmental sources of *A. baumannii* is critical to the prevention of nosocomial infections caused by this pathogen.

**FIGURE 1:**
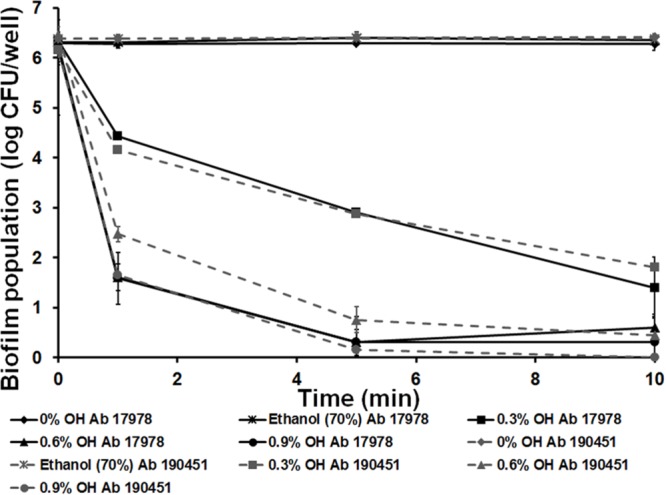
**Inactivation of *Acinetobacter baumannii* ATCC 17978 (Ab 17978) and *A. baumannii* 190451 (Ab 190451) biofilm on polystyrene by octenidine hydrochloride.** Duplicate samples were used for each treatment, and the experiment was replicated three times. Data are represented as mean ± SEM.

Previously, Amalaradjou and Venkitanarayanan (2014) demonstrated the rapid antibiofilm effect of OH against other antibiotic resistant nosocomial pathogens, namely Methicillin-resistant and Vancomycin-resistant *S. aureus*. The present study investigated the antibiofilm efficacy of OH against *A. baumannii*, where the results indicated that OH was effective in rapidly inactivating *A. baumannii* biofilm on all three matrices, namely polystyrene, stainless steel and urinary catheters in the presence and absence of serum protein. OH was equally effective against *A. baumannii* ATCC 17198 (drug susceptible strain) and 190451 (multidrug resistant strain) biofilms. Further, OH retained its antibiofilm efficacy and resulted in a similar inactivation of the biofilm on all matrices, as observed in the absence of protein (data not shown). The effect of OH on *Acinetobacter* biofilms on microtiter plates is shown in **Figure 1**. The treatment of polystyrene surface with 0.6% (10 mM) and 0.9% (15 mM) OH resulted in a reduction in biofilm associated *A. baumannii* population by greater than 5 log CFU/well after 10 and 5 min of exposure, respectively. However, control and ethanol had no significant effect on the biofilm population (*P* > 0.05).

**FIGURE 2:**
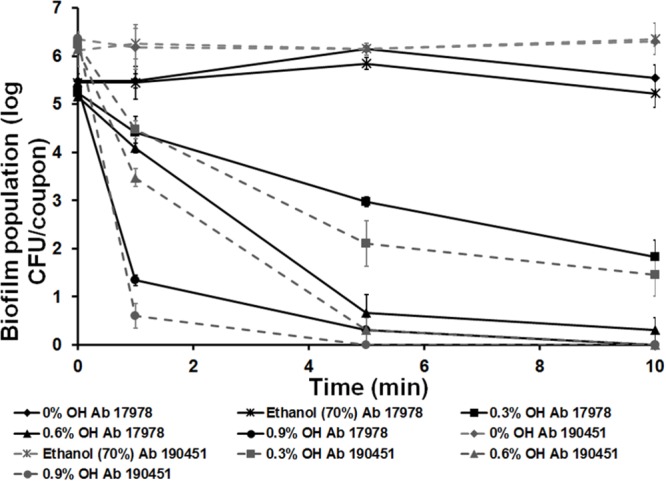
**Inactivation of *A. baumannii* ATCC 17978 (Ab 17978) and *A. baumannii* 190451 (Ab 190451) biofilm on stainless steel by octenidine hydrochloride.** Duplicate samples were used for each treatment, and the experiment was replicated three times. Data are represented as mean ± SEM.

Since *A. baumannii* is capable of forming biofilms on abiotic surfaces such as hospital environments, including stainless implants (Orsinger-Jacobsen et al., 2013), we investigated the antibiofilm effect of OH on stainless steel matrix. It can be observed from **Figure 2** that 0.6% (10 mM) and 0.9% (15 mM) OH decreased *A. baumannii* biofilm to undetectable levels by 10 and 5 min, respectively. A concentration dependent antibiofilm effect was observed with 0.9% (15 mM) OH being more effective than 0.6% (10 mM) and 0.3% (5 mM) OH (*P* < 0.05). As observed with the microtiter assay, the use of ethanol did not exert any antibiofilm effect, with ∼6 log CFU/coupon of biofilm associated bacteria recovered at the end of the study.

In addition to wound infections, *A. baumannii* has been implicated in medical device associated infections, particularly those involving indwelling urinary catheters and central venous catheters (Djeribi et al., 2012; Cobrado et al., 2013). Bacterial biofilms can colonize the inner surface of indwelling urinary catheters and lead to CAUTIs (Djeribi et al., 2012). These biofilms pose a significant threat to patients who have an imperative need for indwelling medical devices. It also makes treatment difficult and in some cases ineffective due to the continuous presence of planktonic bacteria that result from biofilm formation and shedding (Djeribi et al., 2012). Several preventive measures and treatment strategies have been explored to control catheter related infections, including catheter antimicrobial coating (Saint et al., 2000; Jamal et al., 2014), insertion site disinfection (Maki et al., 1991) and antimicrobial catheter lock solution (Shah et al., 2002). A few approaches have had some success, but CAUTI remains a concern worldwide, highlighting the necessity for effective approaches to control *A. baumannii* associated CAUTI. These approaches should be safe and capable of rapidly inactivating existing biofilms on catheters.

**FIGURE 3:**
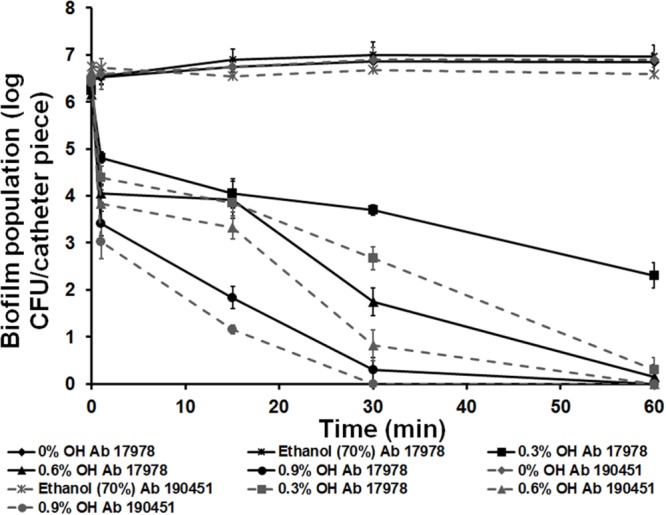
**Inactivation of *A. baumannii* ATCC 17978 (Ab 17978) and *A. baumannii* 190451 (Ab 190451) biofilm in urinary catheters by octenidine hydrochloride.** Duplicate samples were used for each treatment, and the experiment was replicated three times. Data are represented as mean ± SEM.

Besides inactivating *Acinetobacter* biofilms on polystyrene and stainless steel matrix, OH was effective in eliminating biofilms formed on urinary catheters. As a catheter lock solution constituent, 0.6% (10 mM) and 0.9% (15 mM) OH completely inactivated *A. baumannii* biofilms by 60 and 30 min of treatment, respectively (**Figure 3**). Treatment of catheters with 0.9% (15 mM) OH reduced the biofilm population by 3 log CFU/catheter piece almost immediately after exposure, with complete inactivation observed at 30 min. Biofilm counts on control catheters remained ∼6.0 log CFU/catheter piece throughout the experiment. However, all OH concentrations significantly reduced biofilm population to below detection levels by 60 min of exposure. These results indicate that OH is more effective in inactivating *A. baumannii* biofilm in comparison to ethanol (70%), a commonly used disinfectant.

In addition to ethanol, hospitals may also employ Lysol (Alkyl dimethyl ammonium chloride), hydrogen peroxide and hypochlorite (household bleach). Orsinger-Jacobsen et al. (2013) evaluated the efficacy of commonly used hospital disinfectants (Lysol, ethanol, hydrogen peroxide, and hypochlorite) on their ability to inhibit *A. baumannii* biofilms on stainless steel. They observed that all of the tested strains were 100% susceptible to 3.125% hypochlorite, with complete inactivation of the biofilm after 1 min of exposure. However, in the present study we observed that OH at much lower concentration (0.9%) could rapidly inactivate *A. baumannii* biofilm not only on stainless steel but also on polystyrene and urinary catheters. **Table 1** summarizes the anti-biofilm efficacy of OH against *A. baumannii* in comparison with other disinfectants.

**Table 1:** Antimicrobial efficacy of octenidine hydrochloride against *Acinetobacter baumannii* biofilm in comparison to commonly used disinfectants.

Disinfectant	Concentration	Matrix	Log reduction in biofilm population (log CFU)	Time required for log reduction (min)	Reference
Alkyl dimethyl ammonium chloride (Lysol)	37.5%	Stainless steel	No reduction observed	≥20	Orsinger-Jacobsen et al., 2013
Hydrogen peroxide	6.25%	Stainless steel	No reduction observed	≥20	Orsinger-Jacobsen et al., 2013
Hypochlorite	3.125%	Stainless steel	≥5	≥1	Orsinger-Jacobsen et al., 2013
Quaternary ammonium compounds	15%	Polystyrene	No log reduction observed	≥60	Ramos and Alonso, 2013
Octenidine hydrochloride	0.9 %	Polystyrene Stainless steel Foley’s catheter	≥6 ≥6 ≥6	5–10 5–10 30–60	This study
Ethanol	70%	Polystyrene Stainless steel Foley’s catheter	No reduction Observed on all Matrices tested	≥10 ≥10 ≥60	This study

**FIGURE 4:**
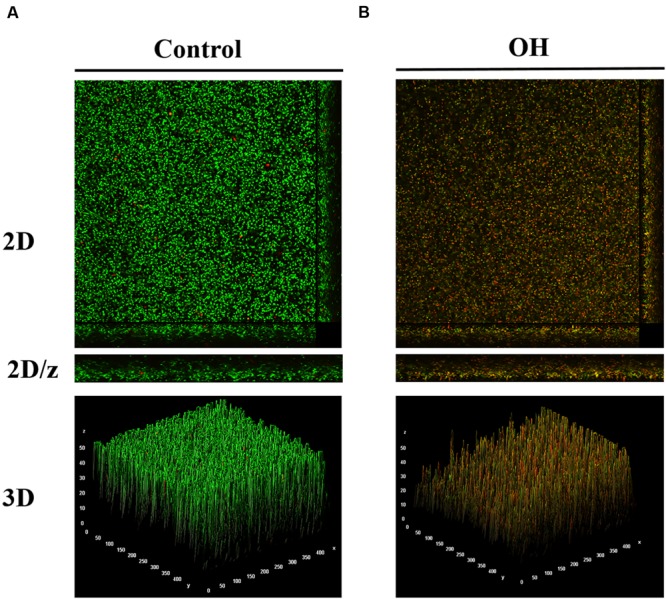
**Confocal microscopy of *A. baumannii* ATCC 17978 biofilm without treatment **(A)** and after treatment with octenidine hydrochloride (B)**.

**FIGURE 5:**
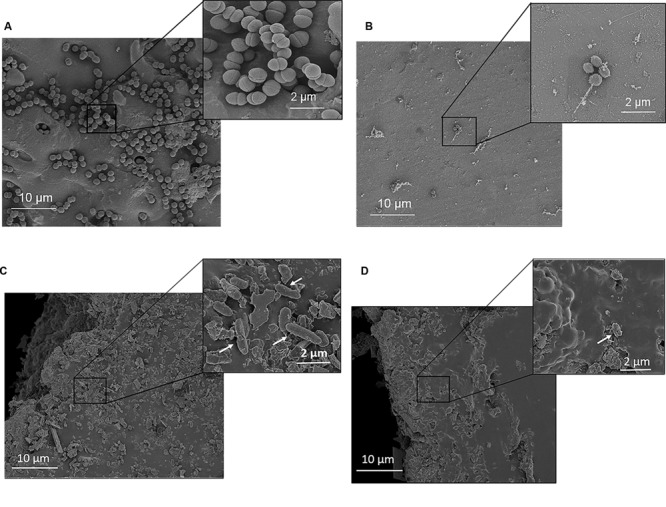
**Ultrastructural images of *A. baumannii* ATCC 17978 biofilm on stainless steel coupons and urinary catheters. (A)** Coupons without treatment, **(B)** coupons after OH treatment, **(C)** catheter without treatment, and **(D)** catheter after OH treatment. Arrows indicate *A. baumannii* cells on the catheter surface.

To investigate the effect of OH on biofilm structure, *A. baumannii* biofilms formed on glass coverslips were analyzed by confocal microscopy. Positive staining using SYTO and Propidium iodide (PI) was used for imaging the biofilms. The confocal images of the negative control samples (**Figure 4A**) with no added OH revealed the formation of a dense biofilm viewed as green cells (live) stained by the SYTO dye, while the image of OH-treated samples (**Figure 4B**) revealed the presence of orange-red cells (stained by PI) indicating the biofilm is composed of dead and dying cells as opposed to the control. Although the biofilm was uniformly distributed over the imaged surface, analysis for % live-dead using ImageJ (Rasband, 1997–2015) revealed that 92% of the biofilm in the OH-treated sample was comprised of dead, non-viable cells as opposed to 100% live in the control. Furthermore, in order to visualize the morphology and ultrastructural features of *A. baumannii* biofilms on stainless steel and catheters, SEM was performed. The SEM images of *A. baumannii* biofilm grown on stainless steel coupons and urinary catheters are depicted in **Figure 5**. These images confirm the presence of biofilm growth on the coupons (**Figure 5A**) and catheter pieces (**Figure 5C**), where the biofilm is visible as a monolayer of individual cells scattered over the matrix surface (**Figure 5**). In addition, analysis of the cross-section and inner surface of the catheter samples revealed that the relative number of bacteria attached to the catheter pieces treated with OH was significantly less compared to the untreated samples (**Figure 5D**). A similar reduction in bacterial population was also observed in the OH-treated coupons (**Figure 5B**). Furthermore, OH treated biofilm bacteria appeared to be dehydrated with evident surface changes (**Figure 5D**).

The antibiofilm effect of OH on *A. baumannii* can be attributed to its binding to the negatively charged bacterial cell envelope, thereby disrupting vital functions of the cell membrane and killing the cell (Brill et al., 2006). It has a high affinity toward cardiolipin, a prominent lipid in bacterial cell membranes, making it selectively lethal to bacterial cells without adversely affecting eukaryotic cells (Stewart and Costerton, 2001). In addition, Al-Doori et al. (2007) reported that repeated exposure of *S. aureus* to OH for up to 3 months did not induce resistance to the compound. Therefore OH could be used as a potential antimicrobial compound in controlling *A. baumannii* biofilms.

## Conclusion

Results from the present study demonstrate the efficacy of OH in rapidly inactivating *A. baumannii* biofilms on polystyrene, stainless steel and urinary catheters in the presence and absence of serum protein. Furthermore, the study demonstrates that OH was equally effective in inactivating biofilms formed by multidrug resistant and drug susceptible *A. baumannii* strains. These results suggest that OH could be used as a sanitizer for hospital surfaces. Additionally, since *A. baumannii* can form biofilms on indwelling catheters, OH can be used as a potential antimicrobial lock solution in catheters. However, further experiments are needed to evaluate the stability and efficacy of OH in comparison with other disinfectants, especially under hospital settings.

## Author Contributions

MA is the corresponding author and primary contact during the manuscript submission, review and publication process. The work was done under the supervision of MA and KV as principal investigators. MA and KV contributed significantly to the design, drafting, revisions, and interpretation of data. MA is the submitting author and accountable for all parts of the work done and questions related to accuracy and integrity of the entire work. AN is the major player in the conception, design, conduct, revision, analysis, and interpretation. DK, MN, and SB contributed to the conduct of different sections of the entire work. All authors have agreed to be accountable to the different parts of the work. The manuscript is being submitted with their final approval for publication.

## Conflict of Interest Statement

The authors declare that the research was conducted in the absence of any commercial or financial relationships that could be construed as a potential conflict of interest.
